# Medical Admonitions

**Published:** 1799-07

**Authors:** James Parkinson


*The press of new matter, in our last Number, prevented us from continuing the analysis of Mr. JAMES PARKINSON's useful work, entitled "Medical Admonitions", of which we gave a short account in our third Number, page* 310; *and which, according to our promise, we now resume.*

THE Author treats of all the diseases and accidents common in this country, in a concise and perspicuous manner; adding such advice and cautions as are best calculated to answer the intentions expressed in the title-page.—As specimens, we select the following passages.

*The malignant ulcerated sore throat,* p.116.

"As the fever increases, grey or ash-coloured spots appear on the uvula, tonsils &c. these extend themselves, in proportion to the violence of the disease; frequently spreading and running one into the other, with the utmost rapidity, the debility becoming excessive, and a continual discharge of a thin acrid humour taking place from the nose and mouth, corroding both the lips and nostrils. As the disease proceeds, the greyish crusts are discovered to be deep gangrenous sloughs; the breath becomes exceedingly offensive, and the disease soon increases to such a degree, unless happily opposed by successful means, as to carry off the patient, sometimes, within the third day after the attack.

From this slight sketch, the domestic practitioner must be apprised of the malignity of this disease, and the rapidity of its progress; and he may also be assured, that he will not always be able to distinguish it, at its first attack, even from the inflammatory sore throat. A mistake here would not only occasion an omission of the proper remedies, but the employment of such means as must necessarily very much expedite a fatal termination."

*The croup, or inflamation of the wind-pipe.* p. 122.

"The exquisite degree of danger which always accompanies this disease; the rapidity with which its symptoms proceed, and the probability of its escaping a sufficiently early detection, will induce me to be rather diffuse in its description; hoping, that parents may thereby be enabled to discover it immediately on its appearance, and be induced to apply for medical aid in the first moments of the disease."

Then follows a very correct enumeration of the symptoms of croup, parcularly those which occur at its commencement; to which we refer our readers.

*Hydrocephalus, or watery head.* p. 440.

"No one, surely, can hesitate for a moment, in believing that the treatment of this melancholy disease ought only to be confided to the most judicious and experienced. I shall, therefore, only mark out those symptom which ought to arouſe the attention of the parent, and occaſion him immediately to call in the moſt powerful aid; and point out ſome circumſtances, by an attention to which this malady may, perhaps, be ſometimes prevented.

"This diſeaſe generally occurs within the firſt ten years of life; ſometimes the complaint comes on ſuddenly, but in general it commences with a ſlow fever; and is indeed accompanied, in its beginning, by ſuch ſymptoms as to render it very likely to be miſtaken for an attack of the ſlow nervous fever. Soon, however, the diſeaſe is rendered more manifeſt, by a diſinclination to employ the muſcles on which voluntary motion depends.

"The arms and legs are moved with reluctance, and the fatigue of preſerving the body in an erect poſture is ſuch, that the patient is always deſirous of being laid down; the pain in the head is more conſtant than in the low nervous fever; and the heavineſs and dullneſs more evident: the pulſe is alſo uſually very ſlow and irregular. As the diſeaſe proceeds, the pulſe becomes quicker; the child's ſenſes and faculties become evidently impared, the ſight particularly fails him, objects appearing exceedingly indiſtinct, and the pupils of the eyes are conſtantly dilated. Towards the cloſe of this melancholy ſcene, the urine and ſtools are paſſed involuntarily; total blindneſs comes on, and a fatal termination takes place, while the patient lies in a comatoſe ſtate, or whilſt agitated with ſevere convulſions.

"I here conclude the taſk allotted me, hoping that, notwithſtanding its imperfections, this little work may prove of real utility. Your candour will, I truſt, prevent you from inferring, from my philippics againſt domeſtic quackery, that it is my wiſh to leſſen the diffuſion of uſeful knowledge. Indeed, on the contrary, I am confident that the beſt and moſt effectual mode of checking the career of empiriciſm would be, by more frequently admitting the ſtudy of anatomy, phiſiology, pathology, and chemiſtry, as part of a liberal education." p. 497.

